# HspB5 Chaperone Structure and Activity Are Modulated by Chemical-Scale Interactions in the ACD Dimer Interface

**DOI:** 10.3390/ijms25010471

**Published:** 2023-12-29

**Authors:** Chenwei Wang, Lilong Teng, Zhiyan Silvia Liu, Aichurok Kamalova, Kathryn A. McMenimen

**Affiliations:** 1 Program in Biochemistry, Mount Holyoke College, South Hadley, MA 01075, USA; wang37c@mtholyoke.edu (C.W.); teng22l@mtholyoke.edu (L.T.); liu38z@mtholyoke.edu (Z.S.L.); 2 Program in Neuroscience and Behavior, Mount Holyoke College, South Hadley, MA 01075, USA; kamal22a@mtholyoke.edu; 3 Department of Chemistry, Mount Holyoke College, South Hadley, MA 01075, USA

**Keywords:** small heat shock proteins, aggregation, chaperone, mutations, disease, protein misfolding

## Abstract

Small heat shock proteins (sHsps) are a family of ATP-independent molecular chaperones that function as “holdases” and prevent protein aggregation due to changes in temperature, pH, or oxidation state. sHsps have a conserved α-crystallin domain (ACD), which forms the dimer building block, flanked by variable N- and C-terminal regions. sHsps populate various oligomeric states as a function of their sequestrase activity, and these dynamic structural features allow the proteins to interact with a plethora of cellular substrates. However, the molecular mechanisms of their dynamic conformational assembly and the interactions with various substrates remains unclear. Therefore, it is important to gain insight into the underlying physicochemical properties that influence sHsp structure in an effort to understand their mechanism(s) of action. We evaluated several disease-relevant mutations, D109A, F113Y, R116C, R120G, and R120C, in the ACD of HspB5 for changes to in vitro chaperone activity relative to that of wildtype. Structural characteristics were also evaluated by ANS fluorescence and CD spectroscopy. Our results indicated that mutation Y113F is an efficient holdase, while D109A and R120G, which are found in patients with myofibrillar myopathy and cataracts, respectively, exhibit a large reduction in holdase activity in a chaperone-like light-scattering assay, which indicated alterations in substrate–sHsp interactions. The extent of the reductions in chaperone activities are different among the mutants and specific to the substrate protein, suggesting that while sHsps are able to interact with many substrates, specific interactions provide selectivity for some substrates compared to others. This work is consistent with a model for chaperone activity where key electrostatic interactions in the sHsp dimer provide structural stability and influence both higher-order sHsp interactions and facilitate interactions with substrate proteins that define chaperone holdase activity.

## 1. Introduction

The cellular milieu is an ever-changing and hostile environment, rendering a substantial fraction of proteins vulnerable to misfolding. Maintaining proteostasis is a delicate balancing act enabled in part by a network of molecular chaperones. In this network are small heat shock proteins (sHsps), essential ATP-independent molecular chaperones that are ubiquitous across life and cell types and are upregulated during the onset of cellular stress [1,2,3,4,5].

**Figure 1:**
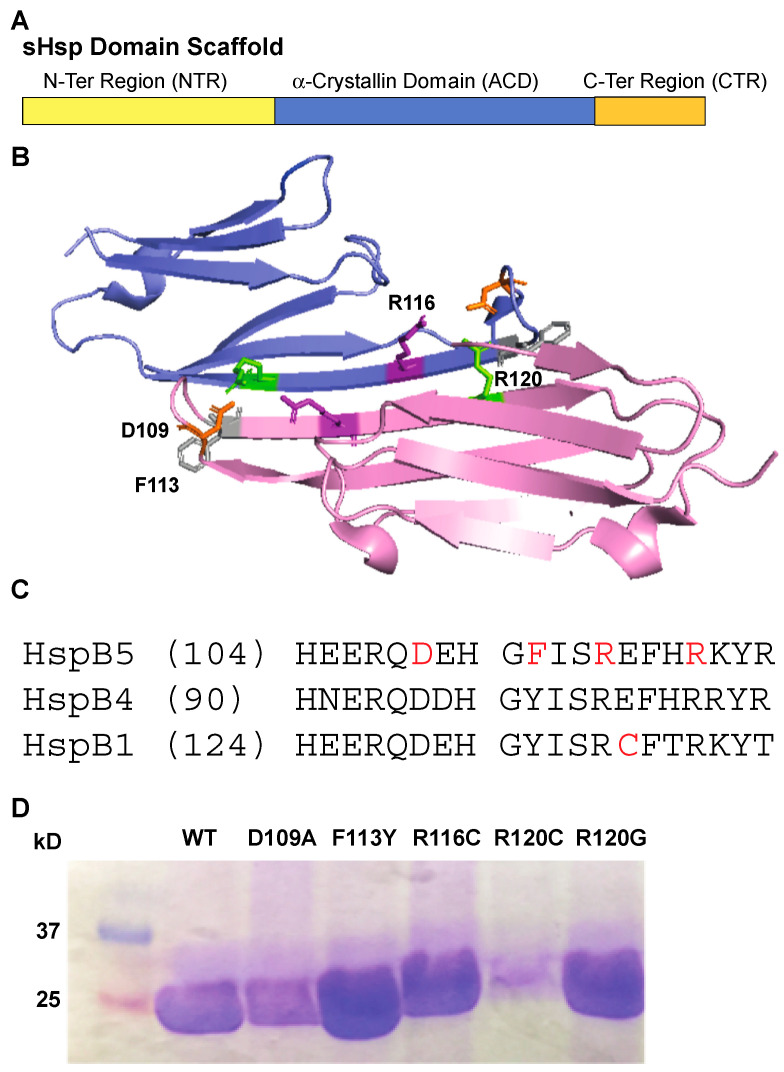
sHsp domain structure and location of ACD mutagenesis within the dimer interface of HspB5 [76]. One monomer is highlighted in blue and a second monomer is highlighted in purple. (**A**) sHsp subunit organization including: N-terminal, α-crystallin domain, and C-terminal domains. (**B**) α-crystallin domain (ACD) structure and point mutation locations along the dimer interface. (**C**) Sequence alignment of the dimer interface resides from three human small heat shock proteins. Residues highlighted in red correspond to mutations in the ACD. (**D**) Coomassie-stained protein gel of purified HspB5 and mutant HspB5 proteins.

There are 10 mammalian sHsps named HspB1–10, which are characterized by low molecular weight (14–43 kDa) [6,7]. sHsps are organized by a tripartite domain architecture where a highly conserved and structured α-crystallin domain (ACD) is flanked by a more plastic and less conserved N-terminal region (NTR) and shorter C-terminal region (CTR) (Figure 1A) [8,9,10,11,12]. The CTR contains a conserved IXI/V motif, which is important for modulating oligomerization dynamics [13,14]. The NTR is involved in broad interactions; including facilitating substrate binding and structural organization of oligomers.

The ACD is approximately 90 amino acids in length, a structural signature of sHsps. The ACD folds into a β-sandwich consisting of two antiparallel β-sheets, with three and four strands each (Figure 1B) [15]. Truncation of the flanking sequences has led to several atomic-resolution structural studies of eukaryotic ACDs that identified a core dimer interface between the β6 + β7 strands of ACD monomers [15,16,17,18]. The interface is decorated with electrostatic interactions (Figure 1C), some of which function as pH-sensitive modulators by altering dimer stability under various conditions [19,20,21,22]. Grooves within the dimer interface contact portions of the N- and C-terminal extensions, contributing to quaternary organization [14,23,24]. Studies of the truncated species have suggested that substrate-binding occurs within the ACD domain. However, evidence also points toward a physiological role for the N-terminal extension and possibly all three domains in retaining in vivo activity [10,25,26,27,28]. It is likely that each domain facilitates sHsp–substrate interactions. 

Dysfunction in sHsps due to mutations or alterations in the proteostasis network is linked to several diseases including cataracts, myopathies, neuropathies, and cancer [1,3,4,23,29,30,31,32,33,34,35,36,37,38,39,40,41,42,43]. sHsps participate in proteostasis by interacting with a large diversity of non-native peptides and proteins to prevent irreversible aggregation [4,44,45,46,47,48]. sHsps function as molecular holdases and sequesterases by binding to unfolded intermediates and isolating them into sHsp/substrate complexes of varying size, stabilizing them from aggregation, degradation, or other destabilizing interactions in the cell [25,26,49,50]. sHsps trap non-native proteins and allow for their transport to ATP-dependent chaperones such as Hsp70 or Hsp90 for further refolding or degradation [16,46,47,51,52]. Recent evidence suggests that sHsps actively form complexes in conjunction with substrate binding, and they are importantly implicated in the disaggregation of non-native substrates. Importantly, there is not a single substrate binding site, and sHsps have the capacity to bind up to equal MW of the client [36,37,38,46,53,54,55,56,57,58].

One of the most significant features of sHsps is their exhibition of a dynamic quaternary structure, characterized by the formation of an ensemble of oligomeric species comprised of varying configurations from 4 to 40 monomers and MW from 40 to 900 kDa. Many studies using truncated and full-length proteins depict the importance of this plasticity [6,17,59,60,61,62,63,64,65,66]. This structural plasticity is a hallmark of sHsps and underlies their physiological chaperone activity [3,54,67]. The hierarchy of sHsp oligomerization begins with dimer formation and continues along a variable trajectory, influenced by substrates, conditions, and other protein–protein interactions in the chaperone network [25,68,69,70,71,72,73]. However, their complex dynamics create technical challenges to studying the structure and mechanism of these proteins, rendering detailed analysis of the structural aspects of sHsps incomplete. Recent studies have indicated that all sHsp domains, the N- and C-terminals along with the ACD, are involved in managing this critical dynamic assembly, which is modulated through a succession of dynamic and weak interactions [8,9,10,11,12,13,14,32,38,74,75].

Disease-related mutations have provided insight into some of the molecular mechanisms of sHsp chaperone activity [13,34,41,42,43,60,77,78]. A well-studied inherited mutation, HspB5 R120G, leads to early-onset cataracts by removing a charged residue from the β6 + β7 strand along the dimer interface, destabilizing the dimer [34,71,79,80]. Maintaining charges at this critical location is insinuated by other disease-related mutations in HspB5, D109A and D109H, which lead to myofibrillar myopathy, cataracts, and multi-systemic pathologies [42,43]. Molecular modeling studies led to a hypothesis that loss of electrostatic interactions at this interface destabilize the interface through β-sheet unfolding. Further in vitro experiments using some of these mutations have identified the propensity of some mutants to form amyloid-like structures and become insoluble under some conditions [39,79,81]. 

Alignment of several human sHsps resulted in identification of charged and hydrophobic residues at the ACD dimer interface, including R116, which are hypothesized to participate in inter-subunit interactions [6,10,14]. Due to the disease-relevant mutations and residues that electrostatic interactions located at the sHsp dimer interface, we designed a series of mutations in HspB5 to examine the relationship between structure alterations and chaperone capacity using several model substrate proteins to explore the implications of altering hydrophobic and electrostatic interactions with disease-specific pathology. These mutations in HspB5 are D109A, D109H, F113Y, R116C, R120C, and R120G (Figure 1C,D). Using model substrates, malate dehydrogenase (MDH), alcohol dehydrogenase (ADH), α-lactalbumin (α-lac), and lysozyme (Lys), which underwent either heat denaturation or chemical denaturation, we compared chaperone capacity of the mutant proteins to that of wildtype HspB5. 

R120G was first identified by Vicart et al. as a cause of desmin-related myopathy [34,79]. It is an autosomal dominant missense mutation that also contributes to congenital cataract [82]. While the in vitro chaperone-like activity of HspB5 R120G has been previously tested using heat-denatured ADH and citrate synthase (CS) as substrate proteins, it is important to replicate the experiments and expand the repertoire of substrates [79], especially those with different denaturation conditions, as unfolding pathways are likely different and chaperone function to these different pathways may vary. Additional substrates will include malate dehydrogenase (MDH), which is similarly denatured by heat, as well as insulin, lysozyme, and α-lactalbumin, which are denatured under reducing conditions.

Previous studies focused on the implications of the R120G mutation on sHsp structure and function and also included the R120C mutation [34,71,82,83]. However, HspB5 R120C proteins from mammalian transfected cells were all insoluble, and downstream experiments, including in vitro chaperone-like activity assays, were aborted [79]. Further studies of arginine to glycine mutations in the dimer interface of a *Drosophila* sHsp note little to modest alterations in structure and function. However, the R116 equivalent residue was not evaluated [84]. Other studies of HspB4 examining arginine to cysteine mutations discovered an altered oligomeric structure and function due to aggregate formation [85]. Given the relevance of electrostatic and hydrophobic interactions that underlie sHsp quaternary structure [86], we hypothesized that F113 may play a role in these interactions based on its location in the dimer. This inspired our study to evaluate the chaperone capacity and structural characteristics of HspB5 mutations, including D109, R120, F113, and R116, that lie in the HspB5 dimer interface. We hypothesized that mutations disrupting the dimer interface would decrease chaperone capacity and alter chaperone structure.

Our studies have revealed that disease-related mutations located in the ACD along the dimer interface exhibited less chaperone capacity compared to that of HspB5 for all model substrates and also exhibited less β-sheet character. Interestingly, the F113Y mutation exhibited more chaperone capacity relative to that of HspB5 for the chemically denatured substrates, insulin and lysozyme, but not with heat-denatured substrates MDH and ADH. We hypothesize that residues in the HspB5 dimer interface contribute to chaperone capacity. Furthermore, HspB5 will have varying capacity for substrates that differ in their mechanism of unfolding.

## 2. Results

### 2.1. Circular Dichroism Spectroscopy Identifies Alterations in Secondary Structure Arising from ACD Mutations 

**Figure 2:**
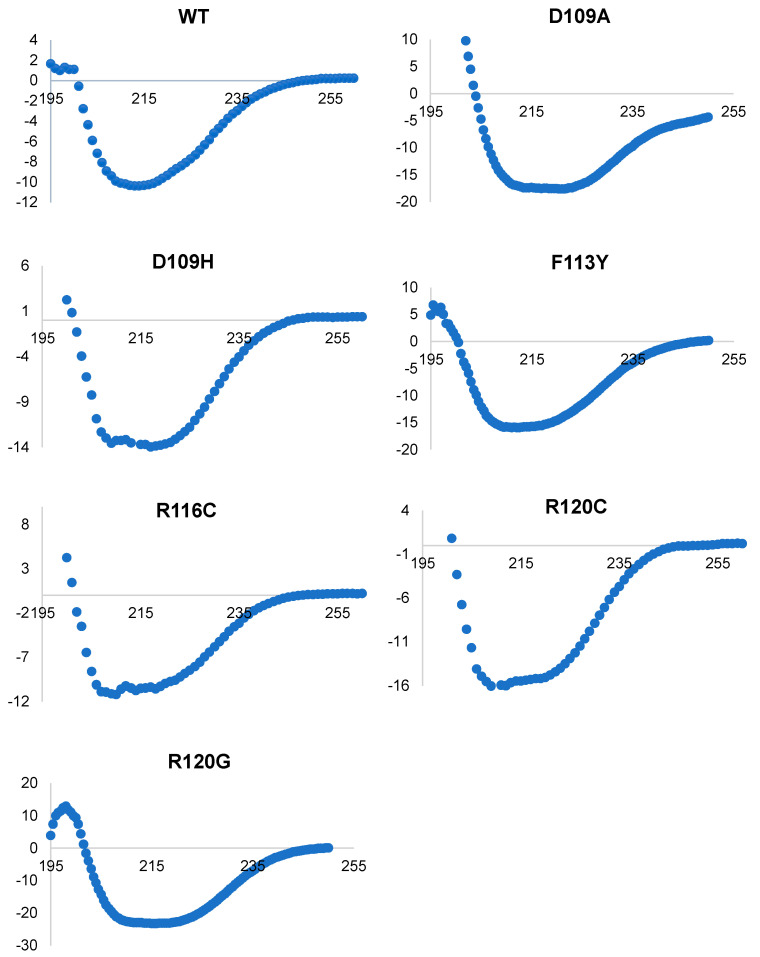
Circular dichroism spectra of HspB5 and dimer interface mutants at 25 °C scanned from 195 nm to 260 nm using a 0.2 cm pathlength. Each sample was performed in triplicate and referenced to PBS under the same conditions.

**Figure 3:**
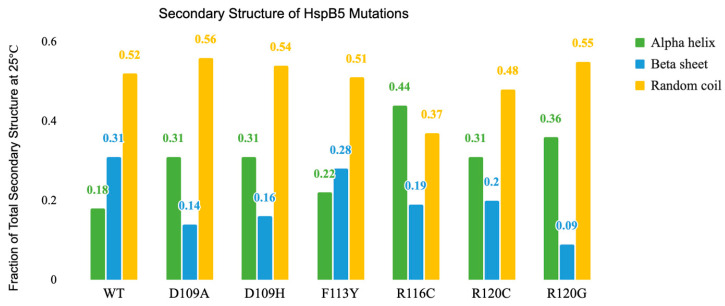
Spectral analysis from DichroWeb of HspB5 and dimer interface mutation. Data were collected at 25 °C from 195 nm to 260 nm. Each sample was referenced to PBS under the same conditions.

The secondary structure of HspB5 and dimer interface mutant proteins was evaluated by circular dichroism (CD) spectroscopy for alterations in secondary structure characteristics. CD measurements were obtained by scanning from 195 nm to 260 nm at 25 °C (Figure 2). Each sample was measured in reference to PBS under the same conditions. Spectral data were input into DichroWeb for analysis of total secondary structure content (Figure 3). HspB5 contains a significant β-sheet character, especially within the highly conserved ACD. Isolated dimer interfaces from several sHsps exhibit characteristic β-sheet character. The dimer interface is formed between β6 and β7. Interestingly, HspB5 exhibited more random coil character compared to β-sheet or α-helix character, as full-length proteins were analyzed in these experiments. Regardless, HspB5 exhibited the most β-sheet character of all evaluated proteins. 

The decline in total protein β-sheet character from all mutations in the interface suggested an alteration to the structure of the α-crystallin domain core (Figure 3). These structural alterations are likely to contribute to differences in the dimer interface as shown in Figure 1. Specifically, F113Y retained the most β-sheet character after HspB5, but also had an increase in α-helical content compared to that of HspB5, likely due to the additional of hydrogen-bonding interactions across the dimer interface with the tyrosine addition. Notably, R120G retained the least of β-sheet characteristics (Figure 2 and Figure 3) and exhibited a decline in chaperone activity. Interestingly, the decrease in overall β-sheet content of each mutant coincided with an increase in α-helix character, but not a change in random coil protein fraction. Since the dimer interface is associated with chaperone activity, disruption to the dimer interface and associated decrease in β-sheet character likely correlates to a decrease in chaperone activity.

Several salt bridges form between β-sheets in the ACD, including R120, previously determined to form a salt bridge with D109. The mutation R120G has been identified as an inherited mutation linked to cataracts and myopathies [34,71,83] and results in decreased β-sheet character, while both random coil and α-helix character are increased as the mutation introduces a small, hydrophobic, and flexible residue at this position (Figure 3). R120C reduces total β-sheet composition compared to that of HspB5; however, the reduction is tempered compared to that with R120G. However, while R120C retains some β-sheet character, there is a large overall increase in α-helical character that suggests the dimer interface and subsequent overall protein structure have been significantly altered.

Mutations at position D109 result in similar structural changes; a decrease in β-sheet character and an increase in α-helical and random coil content. Unsurprisingly, D109A and D109H exhibit very similar secondary structure content. The loss of the salt bridge at the dimer interface likely destabilizes the monomer in addition to altering quaternary structure. The mutation resulting in the highest α-helical content, R116C, also retained substantial β-sheet character while exhibiting a modest decrease in random coil character. 

### 2.2. HspB5 Protein Hydrophobicity Measured by 1,8-ANS Fluorescence 

All HspB5 proteins were evaluated for changes in 1,8 ANS binding and relative fluorescence intensity across three temperatures (25 °C, 50 °C, and 75 °C) from 400 to 540 nm as ANS exhibits sensitive fluorescent changes as a result of changes in protein structure that indicate general protein tertiary structure solvent accessibility [87,88]. An increase in fluorescence intensity, which is sometimes accompanied by blueshift of the fluorescence peak, indicates that the hydrophobic areas on the target proteins are more exposed to a solvent [81]. The dye is quenched by an aqueous solvent, but as it becomes associated with the hydrophobic portions of the unfolded protein, fluorescence is unquenched as the dye is partitioned away from the solvent. Comparison of the ACD mutations to HspB5 would suggest alterations in exposed hydrophobicity due to secondary and tertiary structure changes that expose more or less of the hydrophobic ACD during unfolding. HspB5 proteins were incubated at 25 °C as a control for a folded protein at room temperature, followed by an intermediate temperature of 50 °C, where initial unfolding may occur, but the protein would not have reached Tm as previously recorded [39]. Further heating to 75 °C would lead to unfolding and as a result we would hypothesize fluorescence intensity to increase as ANS has access to the hydrophobic core of HspB5. As previously recorded, exposing hydrophobic regions of sHsp may induce aggregation. Larger aggregates give rise to higher ANS fluorescence intensity, as the dye is no longer quenched by the buffer [89]. Fluorescence intensity of the ANS dye is captured by percent transmittance.

**Figure 4:**
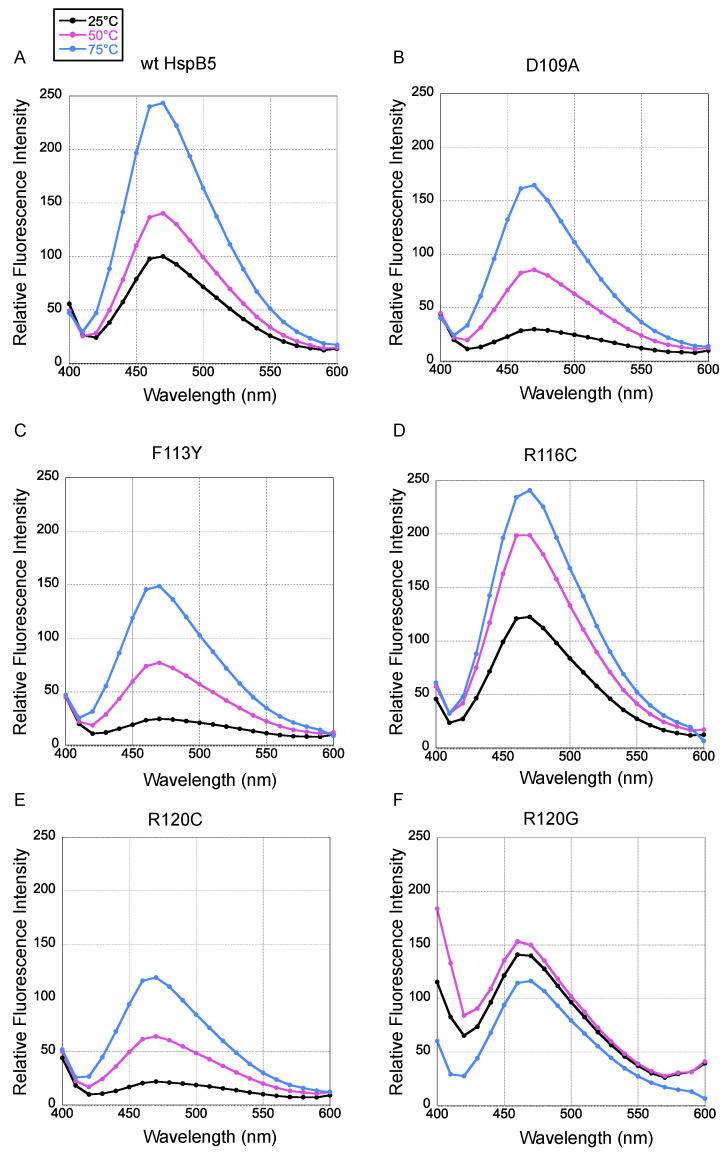
ANS emission spectra of HspB5 and mutant ACD proteins. HspB5 protein or mutants (**A**–**F**) were incubated with ANS at 25 °C, 50 °C, and 75 °C, respectively, for an hour, followed by fluorescence excitation at 475 nm. The emission spectra were captured from 400 nm to 600 nm. Relative fluorescence intensity was calculated relative to HspB5 at 25 °C for all mutations. Raw fluorescence intensity for HspB5 are reported for reference (304 AU ± 40 at 25 °C, 846 AU ± 52 at 50 °C, 1719 AU ± 174 at 75 °C). Relative fluorescence intensity for HspB5 was compared to transmittance in a buffer. Panels A to F correspond to HspB5, D109A, F113Y, R116C, R120C, and R120G proteins, respectively. All data were measured in triplicate.

All fluorescence curves are reported as relative to the transmittance for HspB5 at 25 °C. Disease-relevant mutations (D109A, D109H, F113Y, R116C, and R120C) within the dimer interface of HspB5 were evaluated for deviations in ANS fluorescence intensity compared to that of HspB5 (Figure 4), which indicates changes in the folded state of the proteins compared to HspB5 [65]. Generally, relative fluorescence intensity increased as temperatures were raised for all proteins, except for R120G, where the lowest amount of fluorescence was observed at 50 °C (Figure 4F). At 25 °C and 50 °C, R120G exhibited higher percent transmittance compared to HspB5, but its fluorescence intensity at 75 °C was slightly lower than that for HspB5. Relative fluorescence intensity for R120G was substantially lower than HspB5 at all temperatures (Figure 4F). R120G exhibited a unique trend in fluorescence intensity compared to all other proteins, with low relative values at all three temperatures that did not follow a similar increase in relative percent transmittance in response to an increase in temperature. Peak emission wavelengths were monitored and were measured at 470 nm for all proteins, except for R120G at 25 °C and 50 °C, which was 460 nm, signaling a blueshift at lower temperatures for this mutation. The general instability of R120G has been previously demonstrated, and the ANS data suggest that the folded structure of R120G is more solvent exposed at all temperature conditions, signaling an altered folded state for this mutation relative to wildtype, and likely contributes to its aggregation-prone state [88].

Spectra for the various temperatures were evenly delineated for HspB5, D109A, F113Y, R116C, and R120C, with the spectra merging as the emission wavelength increased toward 600 nm. The spectra for R116C overlapped when the emission wavelength increased from 410 nm to 450 nm at 50 °C and 75 °C, with a larger transition occurring between 25 °C and 50 °C, compared to HspB5. Overall, R116C ANS fluorescence was most similar to that of HspB5, indicating that the solvent exposed the residues and the protein stability of this mutation may be the least perturbed compared to that of wildtype in terms of hydrophobic accessibility by ANS. D109A and F113Y demonstrated a general attenuation in relative fluorescence at all temperatures, which suggested an altered structure compared to that of HspB5 and may indicate that fewer solvent-exposed hydrophobic regions of the protein were available to bind ANS [89].

### 2.3. In Vitro Chaperone-like Activity Assays

**Figure 5:**
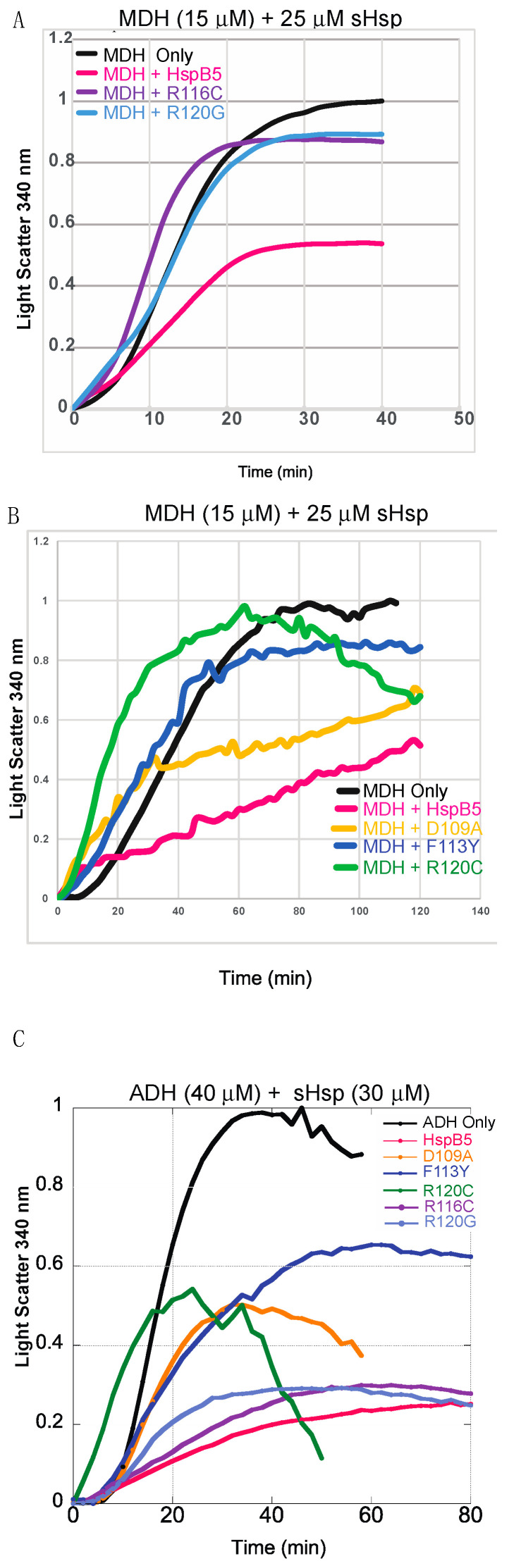
Chaperone aggregation assays for heat-denatured substrates, ADH and MDH. Light scatter for each substrate in the presence of a chaperone at 340 nm was plotted relative to substrate light scatter in the absence of a chaperone. Aggregation was monitored for 90 min at 55 °C. (**A**) MDH only and MDH mixed with HspB5, R116C, and R120G chaperones. Chaperone activity and substrate aggregation were measured at 55 °C for 2 h. (**B**) MDH and HspB5 chaperones. (**C**) ADH and HspB5 chaperones. Chaperone activity and substrate aggregation were measured at 55 °C for 1.5 h for ADH. The data are averages of ≥3 replicates. The data were normalized based on maximum absorbance. In the absence of substrate proteins, chaperones do not aggregate, Figure S1.

sHsps are non-enzymatic chaperones, making their chaperone activity challenging to study compared to other, enzymatic chaperones. Chaperone activity was monitored using in vitro substrate aggregation measured by light scattering. The substrates, when denatured (via heat or chemical denaturant) in the absence of any HspB5, served as positive controls, and their maximum light scatter was reported. Since sHsps have the capacity to interact with a variety of cellular substrates, both heat-denatured and chemically denatured substrates were monitored in order to determine whether differences in chaperone activity may be observed due to the mechanism of substrate denaturation. Reporting light scatter can be variable, as observed for MDH aggregation in Figure 5. Different supply batches of the substrate resulted in varying observed light scatter in the absence of sHsp (Figure 5A,B), which were the respective controls. Generally, the amount of light scatter was the largest in the absence of an sHsp chaperone, indicating a general reduction of and delay in aggregation, as observed in Figure 5. Both malate dehydrogenase (MDH) and alcohol dehydrogenase (ADH) are heat-denatured substrates. After an extended period of heating, the amount of light scattering at 340 nm was reported to decrease for ADH only, ADH with D109A, and ADH with R120C (Figure 5C). This result was consistent with large and visible aggregates that formed, which were insoluble and reduced light scatter by reducing the soluble substrate, which consolidated the substrate.

HspB5 was used as a positive control to establish the baseline chaperone capacity of this small heat shock protein. The amount of light scattering, which corresponds to the extent of aggregation, showed a nearly 50% ± 5% reduction when HspB5 was incubated with ADH (Figure 5). The D109A mutant displayed similar maximum total aggregation relative to HspB5 but had a faster initial aggregate formation compared to the wildtype protein based on the rapid initial increase in light scatter. F113Y showed comparable initial aggregation to D109A and HspB5 when mixed with ADH. However, it had the lowest chaperone capacity and the most light scattering among all the mutants for ADH, but demonstrated more chaperone capacity for MDH. Interestingly, F113Y, was the most similar to HspB5 in relative amounts of secondary structural characteristics (Figure 3) but presented variable chaperone capacity across the heat-denatured substrates (Figure 5). R116C demonstrated a modest reduction in substrate aggregation for MDH ~16% ± 8% and was the only mutant to exhibit more initial light scatter than the substrate alone. However, for ADH, it exhibited an approximate 60% ± 4% reduction in the amount of light scattering relative to the positive control, in total. This indicated a variation in substrate selectivity for each chaperone. Interestingly, R120G demonstrated rapid initial light scatter when incubated with ADH, similar to the substrate by itself, but slowed down rapidly after about 15 min and displayed an approximate 60% ± 3% reduction in the peak light scattering level (Figure 5). Overall, D109A demonstrated substantial chaperone capacity for MDH, similar to HspB5, whereas R116C exhibited ADH chaperone capacity similar to that of HspB5.

For heat-denatured substrates, MDH and ADH, HspB5 was the most effective chaperone. HspB5 exhibited slower aggregation compared to all the mutations, and total chaperone capacity, as observed by maximum light scatter, for both MDH and ADH, was reduced compared to that of all ACD mutations. Specifically, mutations R120C and R120G exhibited faster substrate aggregation and resulted in aggregation that likely involved both the substrate and chaperone, based on an increase in relative aggregation to a similar or slightly increased level relative to substrate only (Figure 5).

To investigate the impact the mechanism of substrate aggregation would have on the chaperone capacity of HspB5 and mutations, three chemically denatured substrates were compared using in vitro chaperone-like assays. The substrates, α-lactalbumin (α-lac), lysozyme (Lys), and insulin, were denatured with DTT. Each substrate in the absence of a chaperone was used as a positive control, and all mutations were compared to HspB5. 

**Figure 6:**
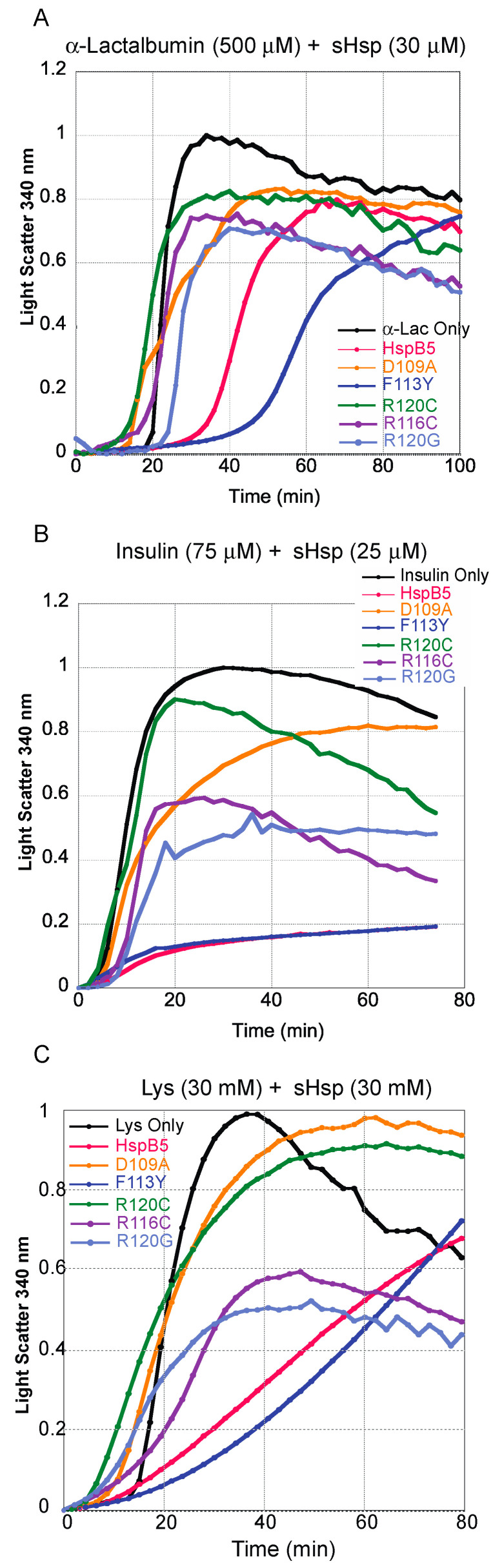
Chaperone capacity of chemically denatured substrates, α-lactalbumin, insulin, and lysozyme. (**A**) α-lactalbumin was incubated with various HspB5 proteins at a 50:3 substrate-to-sHsp ratio, with 7.5 μM DTT at 45 °C. Each curve represents ≥3 replicates. (**B**) Insulin was incubated with various HspB5 proteins at a 5:1 substrate-to-sHsp ratio, with 7.5 μM DTT at 42 °C. The amount of aggregation at time 0 was normalized to 0 with respect to the averaged maximum absorbance of the respective substrates. (**C**) Lysozyme was incubated with various HspB5 proteins at a 1:1 substrate-to-sHsp ratio, with 7.5 μM DTT at 42 °C. Data normalization was performed as described with reference to a buffer in the absence of protein.

Similar trends were observed for all of the chaperones with each chemically denatured substrate; however, the extent of aggregation and the onset of light scatter varied among mutations and with substrates. Each substrate exhibited the greatest extent of total aggregation in the absence of any chaperone (Figure 6A–C) and served as the positive control. One striking result was the chaperone activity of F113Y, which was similar to that of HspB5 when insulin and lysozyme were substrates (Figure 6B,C) and greater when α-lac was the substrate (Figure 6A). Insulin aggregation was reduced as indicated with both HspB5 and F113Y, which exhibited a more than 50% ± 5% reduction in the peak level of light scattering (Figure 6B). In comparison, the chaperone activity of F113Y was less active and consistent for heat-denatured substrates, MDH and ADH (Figure 5). 

Mutations D109A and R120C exhibited the lowest chaperone activity with light scatter reaching maximum aggregation, similar to the substrate in the absence of a chaperone. While total light scatter was similar in the presence and absence of D109A with heat-denatured substrates, light scatter occurred over a longer timeframe when insulin was the substrate. This suggests that there may have been an initial substrate–chaperone (insulin-D109A) interaction that occurs early in the aggregation process. This was not observed for α-lactalbumin or lysozyme. 

Mutations R116C and R120G demonstrated similar chaperone activity across all three chemically denatured substrates. While total light scatter was reduced in the presence of the sHsp compared to the substrate alone (Figure 6), there was an initial, rapid increase in light scatter compared to that of HspB5 and F113Y, indicating altered chaperone activity for R116C and R120G mutations and intermediate chaperone capacity relative to all mutations. When the chaperone capacity of R116C was compared across substrate denaturation, it was highly variable, as it retained some chaperone capacity for insulin and lysozyme (Figure 6) but may still aggregate with MDH (Figure 5). This suggests that the combination of structural, electrostatic, and chemical-scale changes impact chaperone function for some substrates more than others and may arise from variable binding interactions between the substrate and chaperone. Interestingly, the overall patterns of relative chaperone capacities for each mutant were conserved across all heat-denatured substrates, which suggests there may be different mechanisms of sHsp–substrate interaction that arise based on specific substrate unfolding conditions. 

Overall, disrupting the dimer interface through the deletion of electrostatic interactions or through alterations to hydrogen-bonding interactions reduces chaperone activity for heat-denatured substrates. Interestingly, HspB5 and F113Y are both highly functional chaperones for chemically denatured substrates, with F113Y exhibiting more chaperone activity compared to HspB5. Other mutations found in the dimer interface of HspB5 significantly decrease the chaperone activity for all substrates. This suggests that the dimer interface plays a central role in modulating overall chaperone activity and in early-stage association with the denatured substrate, and the unfolding pathway of the substrate may also contribute to the ability of the chaperone to interact with a substrate. 

## 3. Discussion

**Figure 7:**
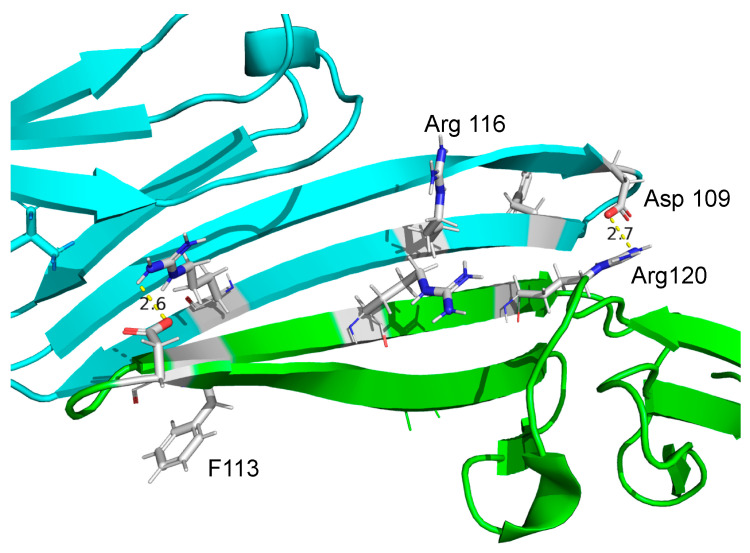
The α-crystallin domain (ACD) dimer interface characterized from HspB5 oligomers using solid state NMR, pdb 2KLR [76]. Residues forming key electrostatic interactions are shown. The salt bridge between D109-R120 is indicated as 2.6–2.7 Å. Other sites of mutagenesis, Arg 116 and F113, are indicated, illustrating their central positions within the interface. Distances were calculated in PyMOL (The PyMOL Molecular Graphics System, Version 2.0 Schrödinger, LLC (New York, NY, USA).).

sHsps are ATP-independent molecular chaperones at the nexus of proteostasis. sHsps interact with misfolded and/or unfolded substrates to prevent aggregation and facilitate transfer to other members of the chaperone network via a holdase function. sHsps also play a role in promoting the disaggregation of some substrates. The mechanism of the sHsp chaperone function has been challenging to untangle due to their dynamic structure heterogeneity [38,90]. sHsps have a characterized structure that spans from monomers, dimers, and small oligomers up to large heterogeneous oligomers, and in vitro chaperone activity has been observed across all structural conformations [24,27,54,91,92]. Structural and functional studies have suggested that sHsp dimers may be the most active chaperone species; however, unraveling the complexity between structure and function for substrates remains challenging [1,5]. The dimer interface in the ACD of all sHsps is relatively similar in primary and secondary structure, with a canonical anti-parallel β6–β7 sheet forming the interface (Figure 1 and Figure 7) [50,93]. The interface is stabilized by a series of salt bridges from backbone interactions and electrostatic interactions mediated by side chains [69,76]. Recent work suggests that the stability of the interface, determined by the number of weak interactions present, contributes to variability in oligomeric structure and can mediate inter-monomer interactions in the absence of the N- and C-terminal domains [34,84,94,95,96]. 

The dimer interface is important not only for making inter-monomer contacts but also in forming interactions with substrates and maintaining chaperone activity while facilitating larger-order oligomeric dynamics as well. Several disease pathologies are linked directly to mutations located in the dimer interface. These mutations suggest that broad destabilization of the dimer interface may impact sHsp–chaperone complex formation and lead to pathologies characterized by chaperone-substrate aggregation. Additionally, sHsps respond to a diverse protein environment, as they are ubiquitous across all cell types and organisms and interact with a broad array of substrate proteins. Therefore, comparing chaperone capacity for different substrates is important for understanding mechanisms of chaperone activity, since chaperones are known to interact to varying degrees with model substrates, and substrate specificity has not been observed [5,97]. However, sHsp chaperone activity is often reported using a single model substrate, which makes comparison of activity for various substrates challenging to interpret. 

In this work, HspB5 and HspB5 dimer interface mutations, R116C, R120C, R120G, F113Y, D109A, and D109H, were evaluated for alterations in structure and function. In order to examine the potential influence of substrate denaturation on chaperone activity, two heat-denatured substrates, MDH and ADH, along with three chemically denatured substrates, α-lactalbumin, insulin, and lysozyme, were evaluated by light scatter at A340, an indicator of protein aggregation (Figure 5 and Figure 6). These mutations were evaluated due to their reported disease-relevance and impact in maintaining electrostatic contacts across the dimer interface [42]. This study is the first to illustrate the reduction in the chaperone capacity of HspB5 D109A, F113Y, R116C, and R120C mutants when interacting with five different substrate proteins in vitro. Our results support a growing body of evidence that the ionic interactions between D109 and R120 are crucial in maintaining the structural and functional integrity of the HspB5 dimer interface [34,71,79,83,98,99]. This work identifies many physiochemical properties along the dimer interface that directly contribute to maintaining its dynamic structure and chaperone activity (Figure 7).

Overall, all mutations except F113Y resulted in alterations in secondary structure with a corresponding decrease in β-sheet character, likely manifesting in altered quaternary structure. These changes generally corresponded with decreased chaperone activity for all substrates; however, the degree of change varied across the mutations. Chaperone capacity of R120G, R120C, and R116C mutations was severely impacted for heat-denatured substrates, but a decline was observed for all substrates. Crystal structure data of the dimer interface indicated that R116 is located in the middle of the b6 strand along the dimer interface. Introducing a cysteine residue allows for potential disulfide cross-linking, stabilizing the dimer (Figure 7). Chaperone capacity was variable for R116C, and secondary structure analysis indicated that this mutation introduced the most α-helix character to the protein compared to all mutations. A decline in heat-induced hydrophobicity changes was reported, suggesting that the mutation may also reduce protein quaternary dynamics. 

R120G is pathologically relevant for cataract formation and has previously been reported to exhibit reduced stability while also demonstrating a propensity to form amyloid-like structures under certain conditions [34,39,71,83,98,99]. R120G exhibits diminished changes in exposed hydrophobic residues upon heating and retains the least β-sheet secondary structure of all mutations evaluated. The chaperone capacity of R120G is reduced. When R120G was mixed with MDH, there was an initial increase in light scatter that may arise from aggregation of both the substrate and chaperone.

Structural information of the HspB5 dimer indicates that F113Y is located at the edge of the β6–β7 sheet (Figure 7). While the mutation introduced a reduction in the accessibility of protein hydrophobic domains based on ANS fluorescence, CD spectroscopy indicated there were very small changes in secondary protein structure compared to that of HspB5. F113Y exhibited enhanced chaperone capacity for all chemically denatured substrates, which suggests that addition of the side chain hydroxyl group resulted in subtle changes in protein structure. Unlike the other mutations we examined, which removed a charge from the dimer interface, the F113Y mutation introduced the potential for new hydrogen bonds forming in the interface. The resulting changes did not prevent favorable substrate–chaperone interactions from forming and instead enhanced sHsp capacity for preventing substrate aggregation of all chemically denatured substrates (Figure 6). Surprisingly, F113Y did not exhibit enhanced chaperone capacity for either heat-denatured substrate. This suggests a possible role for the substrate unfolding pathway in guiding substrate–chaperone interactions and the potential role of hydrogen bonds in making favorable contacts with substrates to augment chaperone capacity.

D109A is a mutation linked to myofibrillar myopathy pathology, and D109H has been associated with cataract and myopathy in five blood-related individuals [42,43]. In dimerized HspB5, D109 makes a salt bridge to R107 on the same monomer and forms hydrogen bonds with Y122 on the opposite monomer [15,71,93,98,100]. Previous work has shown that D80 interacts with both R107 and R120 to stabilize electrostatic repulsions between the arginine residues, and [42] has proposed that in the case of D109A, the R120-D80-R107 interactions were partially preserved, because alanine could not interact with R107 or Y122 [42]. The monomers would consequently have less contact with each other, leading to unfolding or dissociation of the dimer. Based on our results, the presence of a hydrophobic residue within the dimer interface, D109A, likely results in a structure that sequesters this hydrophobic residue in a buried position, relative to HspB5, by decreasing β-sheet character. Accessibility of hydrophobic regions in D109A is slightly reduced compared to that in HspB5, suggesting that the mutation induces a conformational change, observed in both ANS binding and secondary structure determination, due to the increased hydrophobicity of the mutation. Additionally, these structural changes correlate to a reduction in chaperone capacity of D109A compared to HspB5 across all substrates. The extent of reduction was smaller in heat-denatured substrates compared to chemically denatured substrates, suggesting that the unfolding pathway of the substrate may influence the substrate–chaperone interactions and disruption of the physiochemical properties at this position can have pathological consequences.

The highly conserved ACD of sHsps forms critical inter-sHsps contacts at the dimer interface and facilitates higher-order oligomer dynamics [25,69,98]. Additionally, this canonical domain contributes to facilitating protein–protein interactions between sHsps and substrates, while mutations in this domain lead to structural alterations that are linked to several disease pathologies [34,42,79,80,101]. Our studies suggest that several charged side chains in the ACD form ionic interactions that facilitate a β-sheet secondary structure. These residues, R120 and D109, form an essential salt bridge in the dimer. Additionally, ACD mutations display altered forms as indicated by changes to overall protein hydrophobicity measurements at various temperatures. ACD mutations result in altered secondary structure characteristics and demonstrate a decrease in chaperone capacity for both heat-denatured and chemically denatured substrates. According to our results, the extent to which chaperone capacity decreases is dependent on the substrate as well as the sHsp, suggesting that there are likely multiple substrate-interacting regions of the chaperone that facilitate chaperone capacity. Therefore, identifying specific interactions between the sHsp and substrates may provide additional information regarding the mechanisms of sHsp function within the chaperone network. Generally, chaperones interacting with chemically induced denatured substrates exhibited more consistent chaperone capacity compared to the interactions with heat-denatured substrates, which may suggest that regions outside of the sHsp dimer core facilitated at least some of the substrate–chaperone interactions. However, all the mutations result in secondary structure changes that impact chaperone capacity, thus, the sHsp dimer interface forms a structural foundation that facilitates substrate–chaperone contacts, which are critical for maintaining chaperone function. Despite the variability in the mechanism of substrate unfolding, HspB5 is an effective chaperone for all the evaluated substrates. Further studies will evaluate other regions of the sHsp to identify substrate-interacting domains and may provide insight into substrate–chaperone selectivity. Given the complexity of the cellular environment, redundancy in substrate–sHsp interactions may be necessary to support the dynamic substrates and conditions sHsps encounter. 

## 4. Materials and Methods

### 4.1. Materials

Plasmids pMCSG7 for recombinant human HspB5 and R120G mutant were kindly gifted by Dr. Jason Gestwicki (UCSF). QuikChange mutagenesis kit, containing 10× reaction buffer, dNTP, DNA polymerase, and a Quik-change solution, was obtained from Agilent Technologies (Santa Clara, CA, USA). Site-directed mutagenesis primers were synthesized by IDT (Iowa City, IA, USA). HALT protease inhibitor cocktail and 7000 MWCO SnakeSkin^®^ Dialysis Tubing were purchased from Thermo Scientific (Waltham, MA, USA). Precision plus protein standards were purchased from Bio-Rad (Hercules, CA, USA). L-Malate dehydrogenase (MDH) was procured from Roche Molecular Diagnostics (Mannheim, Germany). Lysozyme, α-lactalbumin, insulin, alcohol dehydrogenase (ADH), dithiothreitol (DTT), and 8-Anilino-1-naphthalenesulfonic acid (ANS) ammonium salt were from Sigma Aldrich (St. Louis, MO, USA). 

Mutagenesis was optimized, and forward and reverse primers are listed in Supplemental Information, Table S1. All mutations were confirmed by gene sequencing prior to expression. Protein expression and purification of human HspB5 (αB-crystallin) were performed as previously described [39,66].

### 4.2. Mutagenesis, Protein Expression, and Purification

Site-directed mutagenesis was performed as previously described following protocols and primer design from Quickchange Kit Agilent Technologies (Santa Clara, CA, USA) [66]. Plasmids carrying the desired mutations were transformed into *E. coli* BL21 (DE3) cells and cultured on LB-agar plates containing 100 µg/mL ampicillin. Single colonies were transferred to 5 mL of outgrowth. After 8 h, 500 mL of culture in LB media was inoculated by 1 mL of outgrowth solution. Upon reaching the mid-log growth phase when OD_600_ was between 0.6 and 0.8, protein expression was induced by 0.5 mM isopropyl thio-β-D-thiogalactoside (IPTG) at 25 °C for 5 h. The cells were centrifuged at 11,000 RPM for 15 min and resuspended in a pH 8.0 lysis buffer (20 mM Tris, 100 mM NaCl, 6 M urea, 5 mM β mercaptoethanol, 15 mM imidazole) with a 10 ∝L protease inhibitor cocktail. Sonication of resuspended cells at 70% for 30 s was repeated 4 times with 30 s breaks between each round. The lysed cells were centrifuged at 11,000 RPM for 45 min. The resulting proteins were dialyzed into a pH 7.4 PBS buffer (25 mM sodium phosphate and 100 mM sodium chloride) for 12 h. The proteins were purified with size exclusion chromatography using a Superose 6 Increase 30/100 GL column on AKTA Pure 25 (GE Healthcare Life Sciences, Pittsburgh, PA, USA). The column was calibrated using protein standards (BioRad, Hercules, CA, USA) containing thyroglobulin (669 kDa), γ-globulin (158 kDa), ovalbumin (44 kDa), myoglobin (17 kDa), and vitamin B12 (1.35 kDa) to generate a standard curve. The flow rate was 0.5 mL/min, and the samples were eluted in pH 7.4 PBS at 4 °C. Protein concentrations were determined using a Thermo Scientific (Waltham, MA, USA) BCA assay kit and protein purification by SDS-PAGE gels. Protein gels were stained with Coomassie Brilliant Blue. Purified protein stocks were stored at −20 °C.

### 4.3. ANS Binding Analysis

ANS was dissolved in DMSO for 20 mM stock solutions. Then, 15 µM of HspB5 and each mutant was mixed with 100 µM of ANS diluted in pH 7.4 PBS for a total volume of 300 μL. The solutions were incubated at 25 °C, 50 °C, and 75 °C heat blocks for 1 h, and fluorescence was measured in a 96-well, black, flat bottom, polypropylene plate (Fisherbrand, Hampton, NH, USA) on an M5e multi-mode plate reader. The solutions were excited at 375 nm, and emission intensities were measured from 400 to 600 nm. All values measured in arbitrary units were normalized to the highest value for ANS binding to HspB5 at the respective temperatures.

### 4.4. In Vitro Chaperone-like Activity Assays 

The effect of mutations on heat-denatured substrate proteins was determined by mixing 65.9 ng/µL MDH or 40 mM (5.87 µg/µL) ADH with various HspB5 mutants, wild-type HspB5 as a positive control, or an equivalent volume of pH 7.4 PBS buffer as a negative control. The solutions with a total volume of 100 µL per well were incubated at 55 °C for 1.5 h (ADH) or 2 h (MDH) on a 96-well, transparent, flat-bottom, polypropylene plate (Fisher brand). Absorbance at 340 nm was monitored on an M5e multi-mode plate reader (Molecular Devices, San Jose, CA, USA) at 2 min intervals with 5 s of shaking before each read. Dithiothreitol (DTT)-induced aggregation of 30 µM lysozyme, 75 µM insulin, and 500 µM α-lactalbumin was similarly monitored. Substrates were mixed with 25 mM DTT. Lysozyme and insulin were incubated with HspB5 proteins at 45 °C for 75 min, while the extent of aggregation for α-lactalbumin was monitored at 42 °C for 2.5 h. The absorbance, in arbitrary units, for at least triplicates of each substrate and mutation combination was averaged. The readings were normalized based on maximum absorbance. The amount of aggregation was subtracted by the minimum absorbance and normalized with respect to the averaged maximum absorbance of the respective reactions. Each curve represents ≥3 replicates with % ± SD reported. 

### 4.5. Circular Dichroism Spectroscopy

Protein samples of 0.2 mg/mL were loaded into high-transparency quartz cuvettes with a path length 0.2 cm. PBS baseline was scanned from 195 nm to 260 nm, and CD spectra were collected at room temperature using a JASCO J-1500 spectrophotometer (Oklahoma City, OK, USA). Unfolding was monitored at 216 nm, and the average of at least three independent experiments was reported. The samples were subjected to an increasing temperature from 25 °C to 85 °C at a temperature rate of 1 °C/min, and the samples were measured every 10 °C.

Spectral data were input into DichroWeb (https://dichroweb.cryst.bbk.ac.uk/home.shtml) for data analysis with initial DichroWeb access 10 July 2019.

## Data Availability

The data presented in this study are available in the article.

## Supplementary Materials

The following supporting information can be downloaded at: https://www.mdpi.com/article/10.3390/ijms25010471/s1.
