# Supplementary material for: HspB5 Chaperone Structure and Activity Are Modulated by Chemical-Scale Interactions in the ACD Dimer Interface
Source: Int J Mol Sci. 2023 Dec 29;25(1):471. doi: 10.3390/ijms25010471 (PMC10778692; doi:10.3390/ijms25010471)
Supplement: Supplementary file 1 [file ijms-25-00471-s001.zip › ijms-2755246-supplementary.pdf]

Article

HspB5 Chaperone Structure and Activity are Modulated by Chemical-Scale Interactions in the ACD Dimer Interface

Chenwei Wang [1], Lilong Teng [1], Silvia Liu[1], Aichurok Kamalova [2], and Kathryn A. McMenimen [1,2,3]*

[1] Program in Biochemistry, Mount Holyoke College; wang37c@mtholyoke.edu, teng22l@mtholyoke.edu, liu38z@mtholyoke.edu
[2] Program in Neuroscience and Behavior; kamal22a@mtholyoke.edu,
[3] Department of Chemistry, Mount Holyoke College; kamcmeni@mtholyoke.edu

* Correspondence: kamcmeni@mtholyoke.edu; Tel.: (01-413-538-3375)

Supporting Data

Table S1. Site-directed mutagenesis primers for human HspB5.

Mutations	Primers	
D109A	Forward	5' aaa cat gaa gag cgc cag gct gaa cat ggt ttc atc 3'
	Reverse	5' gga gat gaa acc atg ttc agc ctg gcg ctc ttc atg 3'
D109H	Forward	5' aaa cat gaa gag cgc cag cat gaa cat ggt ttc atc 3'
	Reverse	5' gga gat gaa acc atg ttc atg ctg gcg ctc ttc atg 3'
F113Y	Forward	5' cgc cag gat gaa cat ggt tac atc tcc agg gag ttc 3'
	Reverse	5' gtg gaa ctc cct gga gat gta acc atg ttc atc ctg 3'
R116C	Forward	5' gaa cat ggt ttc atc tcc tgc gag ttc 3'
	Reverse	5' gta ttt cct gtg gaa ctc gca gga gat 3'
R120C	Forward	5' atc tcc agg gag ttc cac tgc aaa ta 3'
	Reverse	5' cat cag ctg gga tcc ggt att tgc ag 3'

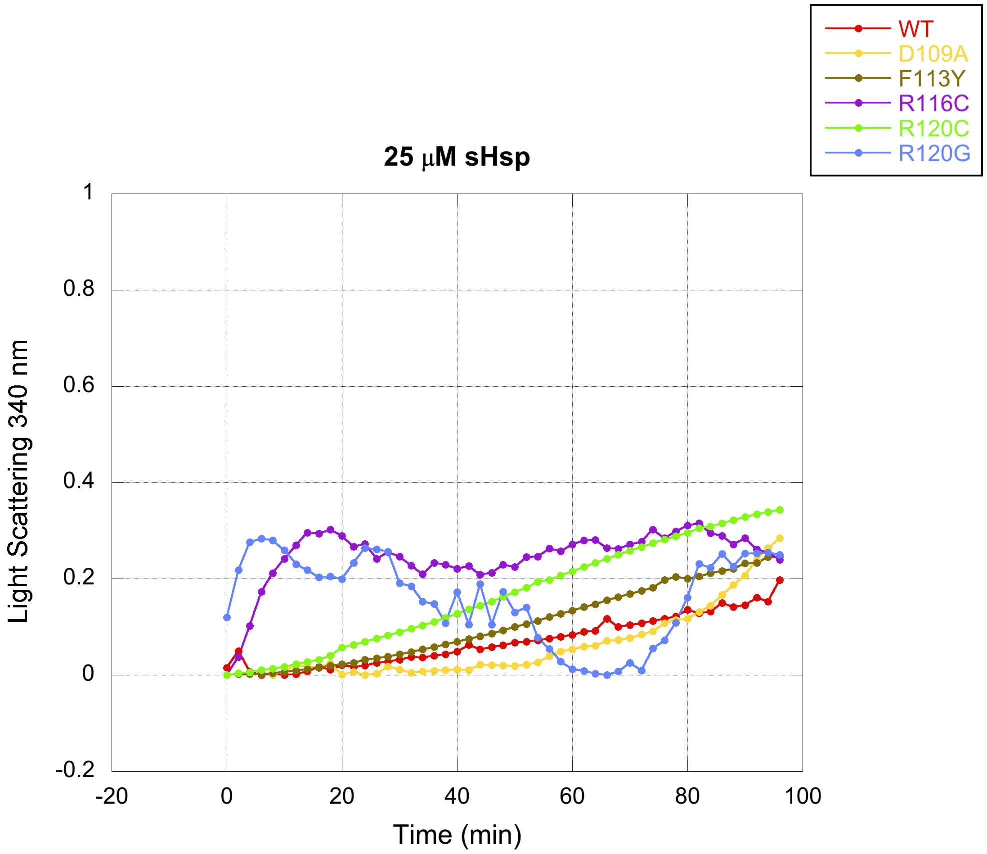

Figure S1. Chaperone-like Assays in the absence of substrate. 25 μM of HspB5 or mutant protein, D109A, F113Y, R116C, R120C, R120G, were mixed with 2.5 μM DTT and samples were heated to 55 °C for the duration of the experiment. Light scatter at 340 nm was monitored. All samples were compared to 1x PBS as a reference. Each sample was run in triplicate.
